# Identification of candidate regulatory sequences in mammalian 3' UTRs by statistical analysis of oligonucleotide distributions

**DOI:** 10.1186/1471-2105-8-174

**Published:** 2007-05-24

**Authors:** Davide Corà, Ferdinando Di Cunto, Michele Caselle, Paolo Provero

**Affiliations:** 1 Dept of Theoretical Physics, University of Turin and INFN, Turin, Italy; 2 Molecular Biotechnology Center and Dept. of Genetics, Biology and Biochemistry, University of Turin, Italy

## Abstract

**Background:**

3' untranslated regions (3' UTRs) contain binding sites for many regulatory elements, and in particular for microRNAs (miRNAs). The importance of miRNA-mediated post-transcriptional regulation has become increasingly clear in the last few years.

**Results:**

We propose two complementary approaches to the statistical analysis of oligonucleotide frequencies in mammalian 3' UTRs aimed at the identification of candidate binding sites for regulatory elements. The first method is based on the identification of sets of genes characterized by evolutionarily conserved overrepresentation of an oligonucleotide. The second method is based on the identification of oligonucleotides showing statistically significant strand asymmetry in their distribution in 3' UTRs.

**Conclusion:**

Both methods are able to identify many previously known binding sites located in 3'UTRs, and in particular seed regions of known miRNAs. Many new candidates are proposed for experimental verification.

## Background

The pathway leading from a gene sequence to the corresponding protein is organized in several steps, all subject to specific regulatory events: from the control of transcription initiation to complex post-translational events that ultimately regulate the fate of the protein product. Increasing evidence indicates that 3' UTRs (3'-untranslated regions) of mRNAs contain different types of short sequence elements playing an important role in the post-transcriptional control of gene expression, regulating mRNA stability, localization and translation efficiency [1].

In particular, a class of small RNAs called micro-RNAs mediate a widespread mechanism of post-transcriptional regulation. Its importance has been clarified in the last few years (reviewed in [2] and [3]). MicroRNAs (miRNAs) are ~ 22nt small non-coding RNAs which negatively regulate gene expression at the post-transcriptional level, in a wide range of organisms. They are involved in many different biological functions, including, in animals, developmental timing, pattern formation and embryogenesis, differentiation and organogenesis, growth control and cell death. MicroRNAs are also known to be relevant to human diseases [4,5].

Mature and active miRNAs are thought to be produced from longer ~ 200nt RNA precursors characterized by imperfect stem-loop structures. These long RNA precursors (pri-miRNAs) are transcribed by RNA polymerase II from particular loci on the genomic DNA, usually called microRNA genes [6-9]. In animals, pri-miRNAs undergo a series of transformations to become mature miRNAs. The latter need to be coupled with a special protein complex called RNA-Induced Silencing Complex (RISC) to become effective as gene regulators [10-13].

Even though the precise mechanism of action of the miRNA/RISC complex is not very well understood, the current paradigm is that miRNAs are able to negatively affect the expression of a target gene via mRNA cleavage or translational repression [14,15], after antisense complementary base-pair matching to specific target sequences in the 3' UTR of the regulated genes. In plants, miRNAs usually have perfect or near perfect complementarity to their mRNA target, whereas in animals the complementarity is restricted to the 5' regions of the miRNA, in particular requiring a "seed" of 7 nucleotides, usually (but not always) from nucleotides 2 to 8 [16-22].

To date, hundreds of miRNAs have been annotated in the genomes of various metazoan organisms together with some of their targets. Each miRNA can regulate between a few and a few hundred genes. In particular, more than 400 miRNA genes have been identified in the human genome and up to one third of the human protein-coding genes is currently believed to be regulated by them [17-21,23-27]. The miRNA binding site is often overrepresented in the 3' UTR sequence of the target gene. Regulation by miRNA is likely a combinatorial mechanism, meaning that a certain mRNA can be under the control of many different miRNAs [23].

miRNAs show interesting evolutionary properties between different species. Indeed, up to one third of the miRNAs discovered in *C. elegans *have a human ortholog. On the other hand, species-specific miRNAs exist and, in particular, it has been established that primates have their own class of miRNA genes [24]. Several computational approaches have been developed in the last four years to investigate this regulatory mechanism (see [28] for a recent review). In particular, computational approaches were suggested for following problems:

• identification of miRNA genes.

• identification of genes regulated by miRNAs.

• description of the regulatory network established by this class of molecules.

Most computational methods proposed to identify miRNA targets are based on some of the following elements:

• evolutionary conservation of miRNAs and their binding sites between species.

• use of the Watson – Crick perfect or imperfect pairing between 3' UTRs and the miRNAs seeds.

• enrichment of miRNA binding sites in 3' UTRs.

• use of RNA secondary structure information.

Important aspects of the effect of miRNAs on the mammalian transcriptome were established in Ref. [29]. In particular the following points will be important for our analysis:

• thousands of mammalian genes are under selective pressure to maintain miRNA binding sites in their 3' UTRs

• evolutionary conservation of the binding sites is a powerful tool to identify biologically relevant sites, not because non-conserved sites are unable to mediate repression, but because they tend to appear in genes which are not co-expressed with the corresponding miRNA

• mRNAs with a miRNA binding site are systematically depleted in the tissues where the miRNA is expressed compared to mRNAs with the same expectation for having sites, taking UTR length and nucleotide composition into account

In this work we present two new methods for the identification of miRNA binding sites, or, more generally, of regulatory sequences located in the 3' UTR of the mRNA. The methods are based on the frequency distribution of oligonucleotides in the 3' UTRs and on evolutionary conservation, and specifically on two working hypotheses:

1. *conserved overrepresentation*: binding sites occur on regulated genes more often than expected by chance, and such overrepresentation is conserved in orthologous genes of closely related species

2. *strand asymmetry*: binding sites appear in 3' UTRs, taken as a whole, more often than their reverse complement. The rationale is that if many genes are under positive selective pressure to maintain binding sites in their 3' UTR, this should cause a global overrepresentation of the binding sites compared to their reverse complements which are not subjected to such positive pressure.

The main novelty of the conserved overrepresentation method is that both the length of 3' UTR sequences and their global nucleotide composition is taken into account when determining whether a certain oligonucleotide is overrepresented in a given 3' UTR. The relative merit of this approach compared to the ones based simply on conserved instances of a motif in 3' UTRs (such as *e.g *Refs. [20] and [21]) will be assessed *a posteriori *by comparing our results to those obtained with such methods.

On the contrary, strand asymmetry has not been used before as a method to identify regulatory elements (in both Refs. [20] and [21] strand asymmetry is referred to differences in conservation scores between a motif and its reverse complement, while our definition refers to a single genome).

More precisely, the assumption we make is the existence of a *statistical correlation *between conserved overrepresentation and strand asymmetry of an oligonucleotide on one hand and its role as a regulatory element on the other. This does not imply that either property is necessary for an oligonucleotide to be a regulatory element. For example, it is known that in several cases a single instance of the binding site (which in most cases is not sufficient to determine statistical overrepresentation) is enough to allow the miRNA to exert its regulatory action. Moreover, since our methods work on fixed words, they rely on perfect matching between miRNA and 3' UTR of the target gene, while it has been shown [30] that "wobbly" G-U pairing does not compromise the regulatory action of the *C. elegans *miRNA *lsy-6 *on one of its targets. On the other hand, *in vivo *experiments [31] on the effectiveness of regulation by a *Drosophila *miRNA showed that (1) mismatches in the seed (positions 2 to 8) significantly reduced the effectiveness of the regulation while (2) the presence of multiple copies of the seed in the 3' UTR strongly increased it. Given these results, and since few miRNA/mRNA interactions have been experimentally validated and studied, while many thousands are believed to exist, we conclude that the only way to test whether these statistical correlations exist is *a posteriori*. Therefore, we will select the oligos displaying conserved overrepresentation and/or strand asymmetry and show that they overlap in a statistically significant way with the binding sites of known regulatory elements acting on the 3' UTR, and in particular with seed regions of known miRNAs.

**Figure 1:**
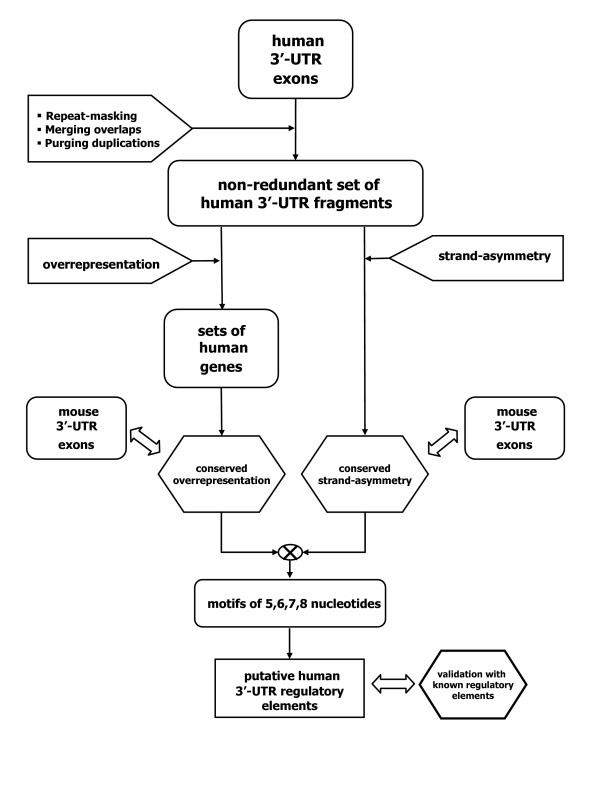
**Flow-chart of the proposed methodology**. Flow-chart of the proposed methodology: from a list of human 3' UTR exons to a list of putative 3' UTR regulatory elements.

The two methods must be considered as complementary, as they are expected to identify different categories of binding sites. A binding site will show strand asymmetry if it is shared by a large number of 3' UTRs. Therefore, we expect the strand asymmetry method to be effective in identifying sites involved in the regulation of 3'-processing, and the binding sites of miRNAs with a large number of target genes. On the other hand, the conserved overrepresentation method is expected to identify those binding sites that tend to appear repeated in the 3' UTR of their targets, and obviously only the ones that are conserved between human and mouse. Since we do not use any information about known miRNAs, the methods we develop are potentially able to identify the binding sites and target genes of both known and unknown miRNAs. A flow-chart of the methodology is depicted in Figure 1.

## Results and Discussion

We analyzed repeat-masked 3' UTR sequences of human and mouse genes using two different methods, both based on the statistical properties of oligonucleotide frequencies:

• *Conserved overrepresentation*. We constructed, separately for human and mouse, sets of genes sharing overrepresented oligonucleotides ("oligos" in the following). We then selected the oligos for which the human and mouse sets contained a statistically significant fraction of orthologous genes.

• *Strand asymmetry*. We identified the oligos showing statistically significant strand asymmetry in their frequency distribution, that is a difference in frequency between the oligo and its reverse complement.

The first method is based on evolutionary conservation without resorting to any alignment procedure: this approach was termed "network-level conservation" in [32] and applied to 3' UTR regulatory elements of flies and worms in [21]. The main difference between our "conserved overrepresentation" method and the one used in [21] is our use of statistical overrepresentation rather then mere presence of an oligonucleotide in the 3' UTR region. On the other hand the "strand asymmetry" approach can be applied to a single genome, and the evolutionary conservation of the results can be checked *a posteriori*. We now turn to a more detailed description of the two methods and of the results found, leaving the more technical details for the "Methods" section.

### Conserved overrepresentation

#### Sets of genes sharing an overrepresented oligo in their 3' UTR region

For each oligonucleotide *w *we constructed, separately for human and mouse, the set of genes such that *w *is overrepresented in the 3' UTR. The analysis was performed for oligo length between 5 and 8, but in the following we will concentrate on 7-mers. The definition of overrepresentation we adopted is that originally introduced in [33] in the context of promoter analysis. Briefly (see Methods for details about statistical procedures), for all *w *we computed the overall frequency *f*(*w*) in all 3' UTR regions, and we selected the genes in which the number of occurrences of *w *is significantly higher than expected based on *f*(*w*).

Statistical significance was determined with a binomial distribution, and a cutoff on P-values equal to 0.01. This procedure is identical to the one we had previously used to identify candidate transcription factor binding sites in upstream sequences [34-36], except that in the present case oligos which are the reverse complement of each other are not counted together, since we are specifically looking for regulatory elements located on single-stranded mRNA. Notice also that, as in the case of upstream regions, no correction for multiple testing was introduced in the construction of the sets; indeed no significance was attributed to overrepresentation by itself (and hence to the sets of genes): only sets showing significant evolutionary conservation between human and mouse, as described below, were selected as candidate binding sites.

#### Conservation of overrepresentation

We then proceeded, for each oligo *w*, to examine the corresponding sets of human and mouse genes looking for enrichment in orthologous genes. Denoting by *S*_*H*_(*w*) and *S*_*M*_(*w*) such sets, we counted how many genes in *S*_*H*_(*w*) had an orthologous gene in *S*_*M*_(*w*), and we determined with the exact Fisher's test whether there was a statistically significant enrichment of pairs of orthologs. The Bonferroni correction for multiple testing was applied to these P-values, taking into account the number of sets compared. Concentrating on oligos of length 7, we obtained 465 oligos with evolutionarily conserved overrepresentation in 3' UTR regions [see Additional file 1]. These are the candidate binding sites obtained by our first method.

### Strand asymmetry

#### Using a Markov chain to construct null-model sequences

We generated a list of oligos *w *significantly more frequent than their reverse complement w¯
 MathType@MTEF@5@5@+=feaafiart1ev1aaatCvAUfKttLearuWrP9MDH5MBPbIqV92AaeXatLxBI9gBaebbnrfifHhDYfgasaacH8akY=wiFfYdH8Gipec8Eeeu0xXdbba9frFj0=OqFfea0dXdd9vqai=hGuQ8kuc9pgc9s8qqaq=dirpe0xb9q8qiLsFr0=vr0=vr0dc8meaabaqaciaacaGaaeqabaqabeGadaaakeaadaqdaaqaaiabdEha3baaaaa@2E34@. In principle also the oligos with significantly *lower *frequency than their reverse complements (which are simply the reverse complements of those included in our list) could be conjectured to be of biological relevance, since many genes are thought to be under selective pressure to avoid developing miRNA binding sites [29]. However these oligos turn out not to be significantly over-represented among seed regions of known miRNAs (data not shown).

The analysis is non trivial due to the peculiar nucleotide frequency distribution of 3' UTR regions, which is reported for our sequences in Table 1 (in agreement with previously reported results [37]). The large difference between the frequencies of A and T implies that the simplest possible way of analyzing oligo strand asymmetry would be misleading: compared to a random sequence, 3' UTR regions would show strong overrepresentation of oligos with many T's compared to their reverse complement, but this would be better explained by the peculiar nucleotide frequencies rather than by functional significance.

A more sophisticated model is therefore needed to take into account the nucleotide frequencies, and possibly other peculiarities of 3' UTR regions, for example in terms of the frequencies of short oligos. The natural solution is to use a Markov chain to construct a model sequence reproducing the oligo frequencies of the sequences under study up to a certain length, and then compare the distribution of longer oligos in the actual sequence to the model sequence. We generated 100 sets of model sequences, separately for human and mouse, reproducing the experimental oligo frequencies up to length 4. Each set of model sequences contained as many sequences as the real set of 3' UTR sequences with the same average length.

**Table 1:** Nucleotide composition of 3' UTR regions.

	Human	Mouse
A	0.2683	0.2638
C	0.2199	0.2237
G	0.2210	0.2254
T	0.2908	0.2871

The base frequencies of 3' UTR regions in human and mouse, excluding the masked repeats.

#### Strand-asymmetric oligos are candidate binding sites

The sequences generated by the Markov chain were used as a null model for the actual sequences: for each oligo *w *we considered the quantity *a*(*w*) = *f*(*w*)- *f*(w¯
 MathType@MTEF@5@5@+=feaafiart1ev1aaatCvAUfKttLearuWrP9MDH5MBPbIqV92AaeXatLxBI9gBaebbnrfifHhDYfgasaacH8akY=wiFfYdH8Gipec8Eeeu0xXdbba9frFj0=OqFfea0dXdd9vqai=hGuQ8kuc9pgc9s8qqaq=dirpe0xb9q8qiLsFr0=vr0=vr0dc8meaabaqaciaacaGaaeqabaqabeGadaaakeaadaqdaaqaaiabdEha3baaaaa@2E34@) where *f*(*w*) is the frequency of *w *and w¯
 MathType@MTEF@5@5@+=feaafiart1ev1aaatCvAUfKttLearuWrP9MDH5MBPbIqV92AaeXatLxBI9gBaebbnrfifHhDYfgasaacH8akY=wiFfYdH8Gipec8Eeeu0xXdbba9frFj0=OqFfea0dXdd9vqai=hGuQ8kuc9pgc9s8qqaq=dirpe0xb9q8qiLsFr0=vr0=vr0dc8meaabaqaciaacaGaaeqabaqabeGadaaakeaadaqdaaqaaiabdEha3baaaaa@2E34@ is its reverse complement. We computed a P-value for the strand asymmetry of *w *based on the assumption of normal distribution of *a*(*w*) in the null model. A Bonferroni correction for multiple testing was applied to these P-values.

**Figure 2:**
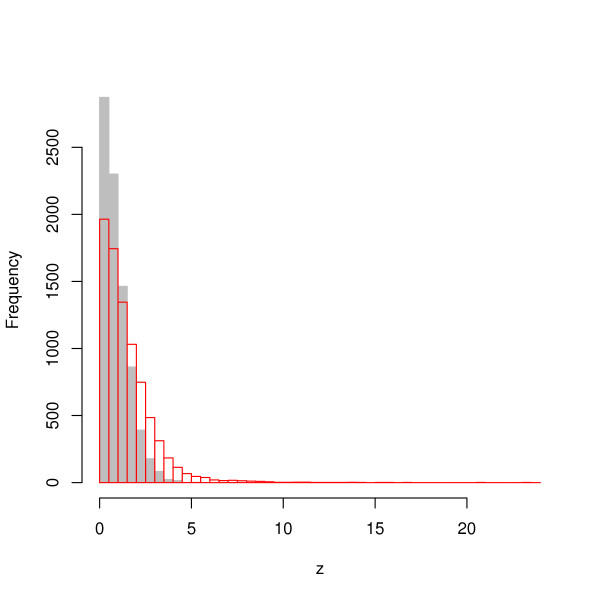
**Distribution of strand-asymmetry *z*-values in 3' UTR and upstream regions**. The distribution of the absolute value of *z*, defined in the text as a measure of strand asymmetry among all possible 7-mers in 3' UTR regions(red) and in 3000 bp upstream of the TSS (grey).

214 oligos of length 7 showed strand asymmetry with Bonferroni-corrected P-value less than 0.01 in the human case, and 139 for the mouse. Of these, 113 were in common (compared to ~ 2 expected by chance): evolutionary conservation was thus recovered *a posteriori*, providing strong support for the biological relevance of the binding sites identified by the method. The lists of the 7-mers showing strand asymmetry in human and mouse are reported in the supplementary material [see Additional files 2 and 3 respectively]. As a control, we compared these results with those obtained by the same analysis on the genomic sequence lying upstream of the transcription start site (TSS) of annotated genes. Since these are not transcribed, we do not expect in this case significant deviations from randomness in the distribution of strand asymmetry. Fig. 2 shows the distribution of the *z*-values calculated on upstream regions of length 3000 bp and the same distribution for 3' UTR regions. As expected, in the case of upstream regions the distribution is much narrower. Indeed only five 7-mers showed significant strand asymmetry: given the difficulty of determining the TSS by automated annotation systems, these cases can probably be explained by the erroneous inclusion of sequence fragments that are actually transcribed.

### Overlap between the candidate binding sites found by the two methods

Of the 214 7-mers identified by strand asymmetry in human, 78 were also identified by conserved overrepresentation (compared to ~ 6 expected by chance in the null hypothesis in which these 7-mers are drawn at random from all 16384 possible 7-mers – P-value 5.6 · 10^-66 ^from Fisher's exact test). Of the 113 7-mers displaying strand asymmetry in both organisms, 59 were also identified by conserved overrepresentation (expected ~ 3.2 – P-value 2.3 · 10^-61^).

It is worth noting explicitly that conserved overrepresentation and strand asymmetry as defined in this work are largely independent properties of *n*-mers. While the overrepresentation of binding sites on target mRNAs is expected to increase the positive selection which is at the origin of strand asymmetry, it is not overrepresentation itself that was used to select the candidate *k*-mers, but its conservation (virtually all *k*-mers are overrepresented according to our definition in at least some genes).

Thus the fact that a significant number of 7-mers were identified by both methods is an important argument in favor of their potential biological relevance. However, as shown below, each method is independently able to identify a statistically significant fraction of seed regions of known human miRNAs and binding sites of other regulatory elements; therefore also oligos identified by only one method can be considered as candidates for experimental verification.

### Comparison with seed regions of known miRNAs

We compared the 7-mers identified by our two methods with a list of 1017 7-mers, representing the seed regions of known human miRNAs. The results of such comparison (*i.e. *the number of positive matches) are shown in Table 2 for the lists of oligos determined by the two methods and various combinations of them [the matching 7-mers and the corresponding miRNAs are listed in Additional files 4 and 5]. P-values were obtained with the hypergeometric distribution, *i.e. *in the null hypothesis in which both the seed regions of known miRNAs and our candidates are represented by randomly chosen 7-mers. The use of this null hypothesis was justified by producing 1000 lists of 1017 7-mers each, randomly generated with a Markov chain reproducing the same dinucleotide distribution as the true seed regions, and computing the mean number of matches with our candidates, which turned out to be in agreement with the prediction of the hypergeometric distribution. The small P-values demonstrate the effectiveness of the method. In the following discussion we will concentrate on the 59 7-mers identified by all methods. This choice maximizes the specificity of the method: indeed we will show that the vast majority of these 7-mers can be recognized as previously known regulatory elements. To increase sensitivity, and thus to obtain larger lists of *new *candidate regulatory elements one can consider the 7-mers identified by each single method separately: the P-values presented in Table 2 demonstrate that the single methods taken separately have significant predictive power.

**Table 2:** Comparison between the results of our computational approach and seed regions of known miRNAs.

Method	7-mers identified	corresponding to known miRNA	expected by chance	P-value
*CO*	465	74	28.9	5.0 · 10^-14^
*SA*	214	41	13.3	7.9 · 10^-11^
*CSA*	113	19	7.01	6.3 · 10^-5^
*CO *∩ *CSA*	59	14	3.66	1.1 · 10^-5^
*CO *∪ *SA*	601	94	37.3	4.8 · 10^-17^

The rows indicate the computational methods as described in the text: *CO *= conserved overrepresentation; *SA *= strand asymmetry; *CSA *= conserved strand asymmetry (oligos displaying strand asymmetry in both human and mouse); *CO*∩*CSA *oligos identified by both *CO *and *CSA*;*CO*∪*SA*: oligos identified by either conserved overrepresentation or strand asymmetry (human) or both. The columns are (1) Number of oligos identified computationally; (2) Number of these matching the seed region of known human miRNAs; (3) number of such matches expected by chance; (4) number of different known human miRNAs putatively binding the oligos (many 7-oligos occur in the seed region of more than one miRNA) (5) P-value from exact Fisher test, taking into account that there are 16384 possible 7-mers 1017 of which are seed regions of known miRNAs.

### Identification of seed regions of known miRNAs and of binding sites of other 3' UTR regulatory elements

While 14 of the 59 7-mers correspond to known miRNA seed regions (shown in Table 3), other known functional elements can be recognized among them (see Table 4). A particularly prominent signal that we would expect to see is the Poly-A signal **AATAAA**. Considering both perfect matches and "side matches" where 5 out of the 6 bases of the PolyA signal match either side of our oligo, 12 of our oligos were thus identified as PolyA signals. Also the variant **ATTAAA **of the Poly-A signal can be recognized in one of the 7-mers.

**Table 3:** 7-mers identified by all methods matching the seed region of known human miRNAs

AGCACAA	hsa-miR-218
CTTTGTA^1^	hsa-miR-524* hsa-miR-520d*
GCACTTT	hsa-miR-520d hsa-miR-93 hsa-miR-106a hsa-miR-520h
	hsa-miR-520a hsa-miR-520e hsa-miR-519b hsa-miR-20a
	hsa-miR-106b hsa-miR-372 hsa-miR-520b hsa-miR-17-5p
	hsa-miR-520g hsa-miR-519c hsa-miR-519d hsa-miR-20b
	hsa-miR-519a hsa-miR-520c hsa-miR-526b* hsa-miR-519e
GGTGCTA	hsa-miR-29c hsa-miR-29b hsa-miR-29a
GTGCAAT	hsa-miR-92b hsa-miR-367 hsa-miR-92 hsa-miR-363 hsa-miR-32
	hsa-miR-25
GTGCCTT	hsa-miR-506 hsa-miR-124a
GTTTACA	hsa-miR-30a-5p hsa-miR-30b hsa-miR-30d hsa-miR-30e-5p hsa-miR-30c
TACTGTA	hsa-miR-101 hsa-miR-199a* hsa-miR-144
TGCAATA	hsa-miR-92b hsa-miR-92 hsa-miR-32
TGCCTTA	hsa-miR-506 hsa-miR-124a
TGTTTAC	hsa-miR-30a-5p hsa-miR-30b hsa-miR-30d hsa-miR-30e-5p
	hsa-miR-30c
TTATATT	hsa-miR-410
TTGTATA^1^	hsa-miR-381
TTTGCAC	hsa-miR-19b hsa-miR-19a

(1) also matches CFI_m _binding site

**Table 4:** 7-mers identified by all methods matching other known regulatory elements

AATAAAC	
AATAAAG	
ATAAAAG	
ATAAAGG	
ATAAAGT	
ATAAATG	
ATTAAAG	PolyA
CAATAAA	
CCAATAA	
CTAATAA	
GAATAAA	
GCAATAA	
TCAATAA	
ATTTAAG	
ATTTATA	ARE
TATTTAT	

GTAAATA	
GTACATA	PUF
TGTAAAT	
TGTACAT	
TGTATAT	
TTGTAAA	

ATTGTAA	
TTTGTAA	PUF, CFI_m_
TTTGTAT	

TTGTATT	CFI_m_

TTTTGTA	CPE, CFI_m_

TTTTATA	
TTTTGTT	
TTTATAA	CPE
TTTATAT	
TTTTTAT	

ATATTTT	
CTATTTT	
GTATTTT	CstF
TATTTTG	
TATTTTT	
TTTTTAA	

Another well known element that we expect to find are the AU-rich elements (ARE), which have been recently linked to miRNAs in triggering mRNA instability [38], with consensus sequences **ATTTA**. We found three of these elements among our entries. Among the most interesting non-miRNA related identifications are nine PUF (Pumilio-FBF protein family) elements (see for instance [39] for a review). The consensus sequence is in this case: **TGTANATA**. We considered both perfect matches and "side matches" defined as in the case of Poly A signals but requiring a match of length 6.

Six of our 7-mers match the CPE element **TTTTAT **allowing for one mismatch. This element is involved in cytoplasmic polyadenylation (see [40] for a review). Note that **TGTAN **is also the consensus sequence for the binding site of the CFI_m _protein, responsible for a non-canonical mechanism of polyA site recognition [41]. Five 7-mers, including three previously recognized as PUF sites and one recognized as a CPE, match this consensus.

More generally T-rich elements can probably be recognized as the binding sites of CstF, a known co-factor of the poly-A site binding protein CPSF [42], or of Fipl [43]. However such elements appear, in our list, almost exclusively in the combinations **TATTTT **or **TTTTNT**. The first of these elements was also identified computationally in Ref. [44], while the second could perhaps be interpreted as a variant CPE element, and is marked as such in the table.

All the known regulatory elements cited above, with consensus sequences shorter than 7, were correctly reproduced by at least one of the methods applied to 5 and 6-mers, with the exception of the alternative polyadenylation site **ATTAAA**. The complete lists of 5-mers and 6-mers identified by the two methods are available in the supplementary material [see Additional files 8, 9, 10 and 11].

#### Comparison with the binding sites of putative miRNAs

The miRNAMap site [45] provides lists of putative miRNAs for many organisms, including human, obtained using RNAz [46], a tool for the prediction of non-coding RNA structures. For 464 of the human putative miRNAs thus obtained, miRNAMap also includes a putative mature miRNA sequence, obtained through a machine learning method. Since these candidate miRNAs were obtained from a computational approach which is completely independent from ours, as it makes no reference to the sequence of the miRNA targets, the comparison of our candidate miRNA binding sites to the seeds of these putative miRNAs provides a further test of our procedure.

We proceeded exactly as we did for known miRNAs, and obtained a list of 939 7-mers to be compared with our candidates. Also in this case we obtained a satisfactory degree of overlapping, detailed in Table 5 [the matching 7-mers and the corresponding putative miRNAs are listed in Additional files 6 and 7]. The overrepresentation of matches survives also if we remove from these 939 7-mers the ones that are also seed regions of known miRNAs (data not shown). Finally Table 6 presents the same comparison made with known and putative miRNA considered together.

**Table 5:** Comparison between the results of our computational approach and seed regions of putative miRNAs listed in miRNAMap [45].

Method	7-mers identified	7-mers corresponding to putative miRNAs	expected by chance	number of putative miRNAs	P-value
*CO*	465	76	26.7	101	6.7 · 10^-17^
*SA*	214	46	12.3	79	3.9 · 10^-15^
*CSA*	113	23	6.48	42	8.8 · 10^-8^
*CO*∩*CSA*	59	15	3.38	31	7.8 · 10^-7^
*CO*∪*SA*	601	98	34.4	135	1.8 · 10^-21^

Same as Table 2 for putative miRNAs. The total number of 7-mers which are seed regions of putative miRNA is 939.

**Table 6:** Comparison between the results of our computational approach and seed regions of known and putative miRNAs

Method	7-mers identified	7-mers corresponding to known or putative miRNAs	expected by chance	number of known or putative miRNAs	P-value
*CO*	465	114	50.3	217	1.5 · 10-^17^
*SA*	214	62	23.1	180	1.6 · 10^-13^
*CSA*	113	30	12.2	96	2.2 · 10^-6^
*CO *∩ *CSA*	59	19	6.38	77	8.0 · 10^-6^
*CO *∪ *SA*	601	149	65.0	285	3.4 · 10^-23^

r Same as Table 2 for known and putative miRNAs together. The total number of 7-mers which are seed regions of known or putative miRNA is 1772.

#### Comparison with putative targets of miR-124 determined in a microarray experiment

Recently an integrated method combining miRNA target predictions and expression profiling was developed in Ref. [47]. In particular the authors published a list of 8 genes downregulated following overexpression of human miR-124. Since the three 7-mers associated to the seed region of this miRNA were identified by our method (GTGCCTT and TGCCTTA by all methods – GCCTTAA by conserved overrepresentation), we checked whether these 8 genes were included in the sets of human genes characterized by the overrepresentation of these 7-mers: indeed five of the eight genes reported (namely SLC16A1, VAMP3, LAMC1, ATP6VOE, ACAA2) do appear in one of the three sets, thus confirming the relevance of oligo overrepresentation in the 3' UTR to the binding of miRNAs.

#### Putative new cis-regulatory elements

Only seven of the 59 7-mers identified by our method could not be associated to any experimentally known binding site or miRNA seed region; these are listed in Table 7. This shows that the use of all the methods combined achieves very high specificity, while clearly sacrificing sensitivity. Higher sensitivity, and thus a higher number of novel candidates, can be obtained by considering the predictions of each method separately.

**Table 7:** 7-mers identified by all methods and not matching experimentally known regulatory elements or miRNA seed regions

AAACTTG
AATCATG
GACCAAA
GTTATTT
TATATGT
TGTGAAT
TTGCCTT

### Comparison with other computational approaches

#### Conserved overrepresentation vs. conserved presence

The first method we proposed, based on conserved overrepresentation, can be compared with the method proposed in Ref. [21], in which the simple presence of a *k*-mer is used instead of statistical overrepresentation. We implemented a method as similar as possible to the one proposed in Ref. [21] on our human and mouse sequences, as detailed in the Methods section. To make the comparison easier we considered the 465 top-ranking 7-mers, that is the same number of 7-mers identified by our conserved overrepresentation method.

Of these 465 7-mers, 77 were recognized as seed regions of known miRNAs (defined in the same way as in the previous section), compared to 74 from the conserved overrepresentation method. Therefore the effectiveness of the two methods is similar, with a slight advantage for the one proposed in Ref. [21]. It is important, however, to notice that the 7-mers identified by the two methods are not the same: only 145 7-mers are in common between the lists determined by the two methods. The two methods have therefore comparable predictive power but give markedly different results, and can thus be expected to complement each other.

It is interesting to look at the distribution in length of the 3' UTR regions of the genes identified by the two methods. Both distributions [see Additional files 12 and 13] are significantly different from the overall length distribution of 3' UTRs, but in different ways: while the 3' UTRs identified by our method show underrepresentation of sequences of length between a few hundred and about 1000 bps, the ones identified by conserved presence show underrepresentation of the shortest sequences. This difference could explain in part the relatively small overlap between the regulatory elements identified by the two methods. In both cases, however, short UTRs tend to be underrepresented, a bias that has a plausible biological explanation: it has been noticed [48] that highly expressed housekeeping genes tend to have short UTRs, a possible explanation for this being [29] that such genes are under selective pressure to avoid acquiring miRNA target sites. Therefore it is plausible to postulate that miRNA targets have relatively longer UTRs. Of course there is no way to determine how much of the bias is due to biological reasons and how much is introduced by the statistical methods.

Finally, note that this comparison should be taken with some caution since in Ref. [21] a correction to the conservation score is introduced based on the length of the 3' UTR sequences, which is not possible to use in our case since it requires the 3' UTRs of orthologous genes to be of equal length.

#### Comparison with the results of Ref. [20]

Ref. [20] describes a computational approach to the identification of regulatory elements in both promoters and 3' UTRs based on comparative genomics. The main differences between this approach and ours are (1) the algorithm of Ref. [20] is based on sequence alignments; (2) it uses information from four different genomes (3) it is based on degenerate motifs rather than fixed words (4) it does not take statistical overrepresentation into account.

To compare the results of [20] to ours we considered the 72 motifs identified in [20] as miRNA-related. These 72 motifs arise from the clustering of 540 different 8-mers. Comparing these 540 8-mers to the binding sites of known miRNAs with the same procedure used for our 7-mers, we found that 89 of them matched the seed region of a known miRNA (*i.e. *an 8-mer appearing within distance 2 from the 5' end of a known miRNA). While the fraction of true positives (89/540 = 0.165) is similar to the one we achieve with conserved overrepresentation (74/465 = 0.159, see Table 2) and lower than the one we obtain with strand asymmetry (41/214 = 0.192), the statistical significance of the results of Ref. [20] is much higher: the same sensitivity corresponds to higher statistical significance since the total number of 8-mers is four times the total number of 7-mers. A higher degree of statistical significance is to be expected since information from four genomes instead of two (or one in the case of strand asymmetry) was used in Ref. [20].

The oligomers identified by our method are significantly different from the ones identified in Ref. [20]: out of 465 (214) 7-mers identified with conserved overrepresentation (strand asymmetry), 140 (69) match one of the 540 8-mers of Ref. [20]. Also in this case, therefore, the two methods can be expected to complement each other.

#### Comparison with the results of Ref. [44]

The authors of Ref. [44] developed a computational method for the identification of a specific class of *cis*-regulatory elements in 3' UTR sequences, namely those involved in the regulation of mRNA polyadenylation. The method radically differs from ours and from the ones discussed above in that it is based on the position of regulatory elements with respect to poly-A sites. Nevertheless, there is a large superposition between their results and ours: most of the hexamers cited in Table 1 of Ref. [44] match one of the 7-mers identified by one or both of our methods. Only for the cis-elements AUE.l, CDE.4 and ADE.4 none of the three top hexamers identified in [44] matched one of our 7-mers.

## Conclusion

We have presented two computational approaches to the identification of cis-regulatory elements located on mammalian 3' UTR regions. Both methods are based on the distribution of oligonucleotides: the first looks for oligonucleotides which are overrepresented in 3' UTR regions of human genes and their mouse orthologs. The second method relies on the identification of oligos displaying statistically significant strand asymmetry, as it should be expected for regulatory elements binding the mRNA of many target genes. The identification of binding sites through strand asymmetry is, to the best of our knowledge, the first *ab initio *method that is based on the statistical analysis of sequences from a single genome.

The effectiveness of the methods is shown by several facts:

• A significant fraction of the candidate binding sites proposed are complementary to the 5' end of known human miRNAs, where the binding to mRNA takes place in most cases

• The same applies to putative miRNAs found by a completely independent computational method [45]

• The two methods, while relying on statistically independent properties ofoligonucleotide distributions, identify many common candidate binding sites

• The candidates identified through strand asymmetry show a remarkable degree of evolutionary conservation even if comparative genomics is not used *a priori*

Taken together, the methods identify 610 7-mers as candidate binding sites. The strong statistical overrepresentation of 7-mers that can be recognized as seed regions of known miRNAs demonstrate their effectiveness. In particular a large majority of the 59 7-mers characterized by conserved overrepresentation and strand asymmetry in both human and mouse are recognizable as experimentally known binding sites, of both miRNA and other *trans*-acting elements, such as those involved in the regulation of polyadenylation.

## Methods

### 3' UTR sequences

3' UTR sequences were obtained from Ensembl [49], version 36 and pre-processed with the following steps (identical for human and mouse):

• Repeat-masked Ensembl exons marked as 3' UTR were downloaded for each protein-coding annotated gene. The masking parameters were left at the default values provided by Ensembl.

• The sequences thus obtained were organized in non-overlapping fragments in which repeat-masked parts were removed. We thus obtained, for each gene, the non-masked part of the 3' UTR as a series of non-overlapping fragments.

• We used BLAST to identify duplicated fragments: we constructed a network of fragments in which two nodes were connected if the BLAST E-value was less than le-40, and we retained only one fragment per each connected component. This procedure guarantees that duplicated sequences appear only once in our sample.

We obtained 45898 human 3' UTR fragments for the human case, and 33728 mouse ones, respectively corresponding to 16800 and 13016 distinct Ensembl ids. The average length of human (mouse) fragments was ~ 364 nt (~ 352), while each gene was on average associated with a set of fragments with total length – 994 (~ 906)nt.

### Oligo overrepresentation

The genes, identified by their Ensembl ids, were first divided into two groups based on the nucleotide composition of their 3' UTR. Indeed the high variability of C/G content among different genomic locations could induce a bias in the statistical analysis of oligo frequencies if not taken into account. Since the classification of genome regions into classes based on their nucleotide composition is still somewhat controversial [50,51], we simply divided our genes, separately for each organism, into two groups (CG-rich and CG-poor) divided by the median CG content of the 3' UTR.

We constructed, separately for each species, the sets *S*(*w*) of genes such that the oligo *w *is overrepresented in the 3' UTR. This was done for all oligos of length between 5 and 8 through the following steps:

• We computed the overall frequency *f*(*w*) as the ratio

f(w)=N(w)N
 MathType@MTEF@5@5@+=feaafiart1ev1aaatCvAUfKttLearuWrP9MDH5MBPbIqV92AaeXatLxBI9gBaebbnrfifHhDYfgasaacH8akY=wiFfYdH8Gipec8Eeeu0xXdbba9frFj0=OqFfea0dXdd9vqai=hGuQ8kuc9pgc9s8qqaq=dirpe0xb9q8qiLsFr0=vr0=vr0dc8meaabaqaciaacaGaaeqabaqabeGadaaakeaacqWGMbGzcqGGOaakcqWG3bWDcqGGPaqkcqGH9aqpdaWcaaqaaiabd6eaojabcIcaOiabdEha3jabcMcaPaqaaiabd6eaobaaaaa@37B3@

where *N*(*w*) is the number of times *w *occurs in the collection of all 3' UTR sequences, and *N *= ∑_*w*_*N*(*w*).

• For each gene *g *let *n*_*g*_(*w*) be the number of occurrences of *w *in the 3' UTR region of *g *(in general made of several fragments, see above). We computed the overrepresentation P-value as

Pg(w)=∑k=ng(w)ng(ngk)f(w)k(1−f(w))ng−k
 MathType@MTEF@5@5@+=feaafiart1ev1aaatCvAUfKttLearuWrP9MDH5MBPbIqV92AaeXatLxBI9gBaebbnrfifHhDYfgasaacH8akY=wiFfYdH8Gipec8Eeeu0xXdbba9frFj0=OqFfea0dXdd9vqai=hGuQ8kuc9pgc9s8qqaq=dirpe0xb9q8qiLsFr0=vr0=vr0dc8meaabaqaciaacaGaaeqabaqabeGadaaakeaacqWGqbaudaWgaaWcbaGaem4zaCgabeaakiabcIcaOiabdEha3jabcMcaPiabg2da9maaqahabaWaaeWaaeaafaqabeGabaaabaGaemOBa42aaSbaaSqaaiabdEgaNbqabaaakeaacqWGRbWAaaaacaGLOaGaayzkaaGaemOzayMaeiikaGIaem4DaCNaeiykaKYaaWbaaSqabeaacqWGRbWAaaGccqGGOaakcqaIXaqmcqGHsislcqWGMbGzcqGGOaakcqWG3bWDcqGGPaqkcqGGPaqkdaahaaWcbeqaaiabd6gaUnaaBaaameaacqWGNbWzaeqaaSGaeyOeI0Iaem4AaSgaaaqaaiabdUgaRjabg2da9iabd6gaUnaaBaaameaacqWGNbWzaeqaaSGaeiikaGIaem4DaCNaeiykaKcabaGaemOBa42aaSbaaWqaaiabdEgaNbqabaaaniabggHiLdaaaa@5AA8@

where

ng=∑wng(w)
 MathType@MTEF@5@5@+=feaafiart1ev1aaatCvAUfKttLearuWrP9MDH5MBPbIqV92AaeXatLxBI9gBaebbnrfifHhDYfgasaacH8akY=wiFfYdH8Gipec8Eeeu0xXdbba9frFj0=OqFfea0dXdd9vqai=hGuQ8kuc9pgc9s8qqaq=dirpe0xb9q8qiLsFr0=vr0=vr0dc8meaabaqaciaacaGaaeqabaqabeGadaaakeaacqWGUbGBdaWgaaWcbaGaem4zaCgabeaakiabg2da9maaqafabaGaemOBa42aaSbaaSqaaiabdEgaNbqabaGccqGGOaakcqWG3bWDcqGGPaqkaSqaaiabdEha3bqab0GaeyyeIuoaaaa@3A59@

is the total number of oligos of the same length as *w *that can be read in the 3' UTR region of *g*. Self-overlapping matches of the same oligo were discarded [33].

• The genes for which *P*_*g*_(*w*) < 0.01 were included in the set *S*(*w*).

The procedure described above was performed separately for CG-rich and CG-poor genes, so that overrepresentation is defined with respect to the appropriate background frequencies. The sets *S*(*w*) computed for CG-rich and CG-poor genes were then joined to obtain a single set *S*(*w*) for each organism.

### Overrepresentation of miRNA binding sites in experimentally known miRNA-3' UTR regulatory interactions

To verify whether our definition of overrepresentation is useful for the identification of miRNA binding sites, we downloaded from miRNAMap [45] a list of 27 experimentally verified instances of such interactions in human (two of the 29 interactions listed in [45] involved a miRNA which is not included in the current version of miRNABase). Each interaction is represented by a miRNA id and a human gene identified by an Ensembl id. For each miRNA involved we defined three possible binding sites as the reverse complement of the 7-mers starting at position 1, 2 and 3 of the 5' end of the mature miRNA sequence as given miRNABase. In 10 of the 27 instances, one of these binding sites was indeed overrepresented, according to our definition, in the 3' UTR sequence of the human gene.

### Conservation of overrepresentation

An oligo *w *has conserved overrepresentation if the sets of genes *S*_*human*_(*w*) and *S*_*mouse*_(*w*) contain a significantly larger number of orthologous genes than expected by chance. Pairs of human-mouse orthologous genes were obtained from Ensembl, selecting only orthologs defined as Unique Blast Reciprocal Hit so as to obtain one-to-one orthology relationships.

Let *M *be the total number of human genes represented in our sequences which have a mouse ortholog. Given an oligo *w *and the set *S*_*human*_(*w*), let *m *be the number of human genes in *S*_*human*_(*w*) which have a mouse ortholog, *N *the number of genes in *S*_*mouse*_(*w*) with a human ortholog, and *n *the number of genes in *S*_*human*_(*w*) with a mouse ortholog in *S*_*mouse*_(*w*). We then compute the P-value

P=∑k=nmF(M,m,N,k)
 MathType@MTEF@5@5@+=feaafiart1ev1aaatCvAUfKttLearuWrP9MDH5MBPbIqV92AaeXatLxBI9gBaebbnrfifHhDYfgasaacH8akY=wiFfYdH8Gipec8Eeeu0xXdbba9frFj0=OqFfea0dXdd9vqai=hGuQ8kuc9pgc9s8qqaq=dirpe0xb9q8qiLsFr0=vr0=vr0dc8meaabaqaciaacaGaaeqabaqabeGadaaakeaacqWGqbaucqGH9aqpdaaeWbqaaiabdAeagjabcIcaOiabd2eanjabcYcaSiabd2gaTjabcYcaSiabd6eaojabcYcaSiabdUgaRjabcMcaPaWcbaGaem4AaSMaeyypa0JaemOBa4gabaGaemyBa0ganiabggHiLdaaaa@40BB@

where

F(M,m,N,k)=(mk)(M−mN−k)(MN)
 MathType@MTEF@5@5@+=feaafiart1ev1aaatCvAUfKttLearuWrP9MDH5MBPbIqV92AaeXatLxBI9gBaebbnrfifHhDYfgasaacH8akY=wiFfYdH8Gipec8Eeeu0xXdbba9frFj0=OqFfea0dXdd9vqai=hGuQ8kuc9pgc9s8qqaq=dirpe0xb9q8qiLsFr0=vr0=vr0dc8meaabaqaciaacaGaaeqabaqabeGadaaakeaacqWGgbGrcqGGOaakcqWGnbqtcqGGSaalcqWGTbqBcqGGSaalcqWGobGtcqGGSaalcqWGRbWAcqGGPaqkcqGH9aqpdaWcaaqaamaabmaabaqbaeqabiqaaaqaaiabd2gaTbqaaiabdUgaRbaaaiaawIcacaGLPaaadaqadaqaauaabeqaceaaaeaacqWGnbqtcqGHsislcqWGTbqBaeaacqWGobGtcqGHsislcqWGRbWAaaaacaGLOaGaayzkaaaabaWaaeWaaeaafaqabeGabaaabaGaemyta0eabaGaemOta4eaaaGaayjkaiaawMcaaaaaaaa@48E3@

Multiple testing was taken into account with the Bonferroni correction, and conserved overrepresentation was defined to be significant when the Bonferroni-corrected P-value was less than 0.01.

### Identification strand-asymmetric oligos

The Markov chain used to construct the model sequences has oligos of length 3 as its states, plus an additional state representing the end of a sequence (we are referring to a simple Markov chain, in which the states are directly accessible by the observer, and not to a hidden Markov model in which they are indirectly accessible through the tokens they emit). The inclusion of the gap state is crucial in correctly reproducing the oligo frequencies derived from model sequences which, as in our case, are heavily fragmented. Transition probabilities between states were computed from the actual sequences (including the transition probability from a 3-mer to the "end of sequence" state). The actual sequences used were the fragments obtained as described above after removing the masked repeats. The initial state was chosen with a probability distribution reproducing the distribution of the initial 3-mers in the actual sequences (that is the transition probabilities from the "end of sequence" state to each 3-mer). Each run of the Markov chain produced therefore a set of sequences reproducing, asymptotically, the following features of the actual set.

• Frequencies of all oligos of length up to 4.

• Frequency distribution of initial and final 3-mers.

• Average sequence length.

The same procedure was applied to determine strand-asymmetric 7-mers in upstream regions to produce Fig. 2. On the set of sequences thus produced we computed, for each oligo *w*, the mean *μ*(*w*) and standard deviation *σ*(*w*) of the quantity

*a*(*w*) = *f*(*w*) - *f*(w¯
 MathType@MTEF@5@5@+=feaafiart1ev1aaatCvAUfKttLearuWrP9MDH5MBPbIqV92AaeXatLxBI9gBaebbnrfifHhDYfgasaacH8akY=wiFfYdH8Gipec8Eeeu0xXdbba9frFj0=OqFfea0dXdd9vqai=hGuQ8kuc9pgc9s8qqaq=dirpe0xb9q8qiLsFr0=vr0=vr0dc8meaabaqaciaacaGaaeqabaqabeGadaaakeaadaqdaaqaaiabdEha3baaaaa@2E34@)

where *f*(*w*) is the frequency of oligo *w *and w¯
 MathType@MTEF@5@5@+=feaafiart1ev1aaatCvAUfKttLearuWrP9MDH5MBPbIqV92AaeXatLxBI9gBaebbnrfifHhDYfgasaacH8akY=wiFfYdH8Gipec8Eeeu0xXdbba9frFj0=OqFfea0dXdd9vqai=hGuQ8kuc9pgc9s8qqaq=dirpe0xb9q8qiLsFr0=vr0=vr0dc8meaabaqaciaacaGaaeqabaqabeGadaaakeaadaqdaaqaaiabdEha3baaaaa@2E34@ is the reverse complement of *w*. The same quantity *a*(*w*) was then computed for the actual sequences, and a *z *value was constructed as

z(w)=a(w)−μ(w)σ(w)
 MathType@MTEF@5@5@+=feaafiart1ev1aaatCvAUfKttLearuWrP9MDH5MBPbIqV92AaeXatLxBI9gBaebbnrfifHhDYfgasaacH8akY=wiFfYdH8Gipec8Eeeu0xXdbba9frFj0=OqFfea0dXdd9vqai=hGuQ8kuc9pgc9s8qqaq=dirpe0xb9q8qiLsFr0=vr0=vr0dc8meaabaqaciaacaGaaeqabaqabeGadaaakeaacqWG6bGEcqGGOaakcqWG3bWDcqGGPaqkcqGH9aqpdaWcaaqaaiabdggaHjabcIcaOiabdEha3jabcMcaPiabgkHiTGGaciab=X7aTjabcIcaOiabdEha3jabcMcaPaqaaiab=n8aZjabcIcaOiabdEha3jabcMcaPaaaaaa@4196@

where *a*(*w*) refers now to the actual sequence. A P-value was finally associated to each oligo *w *assuming a standard normal distribution of the z-values. We retained for further analysis only oligos *w *that are overrepresented with respect to their reverse complement (*z *> 0 and with Bonferroni-corrected P-values less than 0.01).

### Validation with known human miRNA

We downloaded from the miRBase [27] ftp site the file: mature.fa containing the mature sequences of all currently known miRNAs. 454 human miRNAs are annotated in release 8.2, June 2006. To validate our results, we compared the reverse complement of the oligos selected by our algorithm to the 5' end of the mature miRNAs.

Specifically, we extracted three "seeds" for each human mature miRNA included in miRBase, consisting in the three 7-mers starting at the first, second and third nucleotide on the 5' extremity of the miRNA mature sequence. We thus obtained 1017 distinct 7-mers which we consider as seed regions of known miRNAs, to be compared with the 7-mers identified by our method for its validation. This validation procedure requires a perfect Watson-Crick complementarity between the 5' end of the miRNA and the 3' UTR region of its target: while this requirement can be considered too conservative, allowing mismatches and wobbly binding would have increased the number of known miRNA binding sites to include the majority of all possible 7-mers, making the validation of our results meaningless. On the other hand our choice of considering perfect matches only is justified by the fact that these have a significantly lower free energy than imperfect or wobbly ones.

### Conserved overrepresentation vs. conserved presence

We implemented on our mammalian UTR sequences a method based on conserved presence rather than conserved overrepresentation, as similar as possible to the one proposed in Ref. [21]. For each 7-mer we constructed the set of genes containing one or more instances of the 7-mer in their 3' UTR. It should be noted that in our case, as opposed to Ref. [21], the length of the 3' UTR regions of orthologous human and mouse genes are not the same: therefore it is not possible to use the length-dependent correction to the conservation score introduced in Ref. [21].

The human and mouse sets thus constructed were then tested for significant overrepresentation of orthologous genes with the same method used for our overrepresentation sets. As in Ref. [21] we then used the conservation P-values to rank the 7-mers. The 465 highest ranking 7-mers were then compared to the binding sites of known miRNAs with the same procedure adopted for the 465 7-mers identified by conserved overrepresentation.

## Authors' contributions

The four authors jointly conceived and planned the work. Most of the data analysis was performed by D.C. (preparation of the sequences, conserved overrepresentation and comparison with available experimental data) and P.P. (strand asymmetry). All authors participated in writing the manuscript, and all read and approved the final version.

## Supplementary Material

Additional file 1 **List of 7-mers displaying conserved overrepresentation in 3' UTR regions**. For each oligo we report the numbers *m, N *and *n *as defined in the Methods section and the significance of the overrepresentation expressed as – log_10 _of the *P*-value (before Bonferroni correction). We also list for each oligo the *n *human genes with a mouse ortholog in the corresponding mouse set, listing the Ensembl id, gene symbol, Entrez gene id and description. Click here for file

Additional file 2 **7-mers displaying strand asymmetry in human 3' UTR regions**. For each oligo we report the *z*-value as defined in the text Click here for file

Additional file 3 **7-mers displaying strand asymmetry in mouse 3' UTRs**. As in additional file 2. Click here for file

Additional file 4 **7-mers displaying conserved overrepresentation in 3' UTRs: validation with the seed regions of known miRNAs**. For each oligo we report the known human miRNAs with seed region (defined as described in the text) matching the oligo. Click here for file

Additional file 5 **7-mers displaying strand asymmetry in human 3' UTRs: validation with seed regions of known miRNAs**. As in additional file 4. Click here for file

Additional file 8 **List of 6-mers displaying conserved overrepresentation in 3' UTRs**. As in additional file 1. Click here for file

Additional file 9 **List of 6-mers displaying strand asymmetry in human 3' UTRs**. As in additional file 2. Click here for file

Additional file 10 **List of 5-mers displaying conserved overrepresentation in 3' UTRs**. As in additional file 1. Click here for file

Additional file 11 **List of 5-mers displaying conserved overrepresentation in human 3' UTRs**. As in additional file 2. Click here for file

Additional file 6 **7-mers displaying conserved overrepresentation in 3' UTRs: validation with seed regions of putative miRNAs**. As in additional file 4. Click here for file

Additional file 7 **7-mers displaying strand asymmetry in human 3' UTRs: validation with seed regions of putative miRNAs**. As in additional file 4. Click here for file

Additional file 12 **Length distribution of 3' UTRs of the genes conserved overrepresented 7-mers**. 3' UTR length of genes appearing in at least one of 465 sets selected by conserved overrepresentation (red) compared to all genes in our dataset (green). Click here for file

Additional file 13 **Length distribution of 3' UTRs of the genes conserved instances of the 7-mers selected with the method of Ref [21]**. The genes represented in red have at least one conserved instance of one of the 465 hightest-ranking 7-mers identified using the method introduced in [21]. The length distribution of their 3' UTRs is compared to the length distribution of all the genes in the dataset (green). Click here for file
