# Supplementary figures and images for: Identification of candidate regulatory sequences in mammalian 3' UTRs by statistical analysis of oligonucleotide distributions
Source: BMC Bioinformatics. 2007 May 24;8:174. doi: 10.1186/1471-2105-8-174 (PMC1904458; doi:10.1186/1471-2105-8-174)

# All genes vs conserved overrepresentation

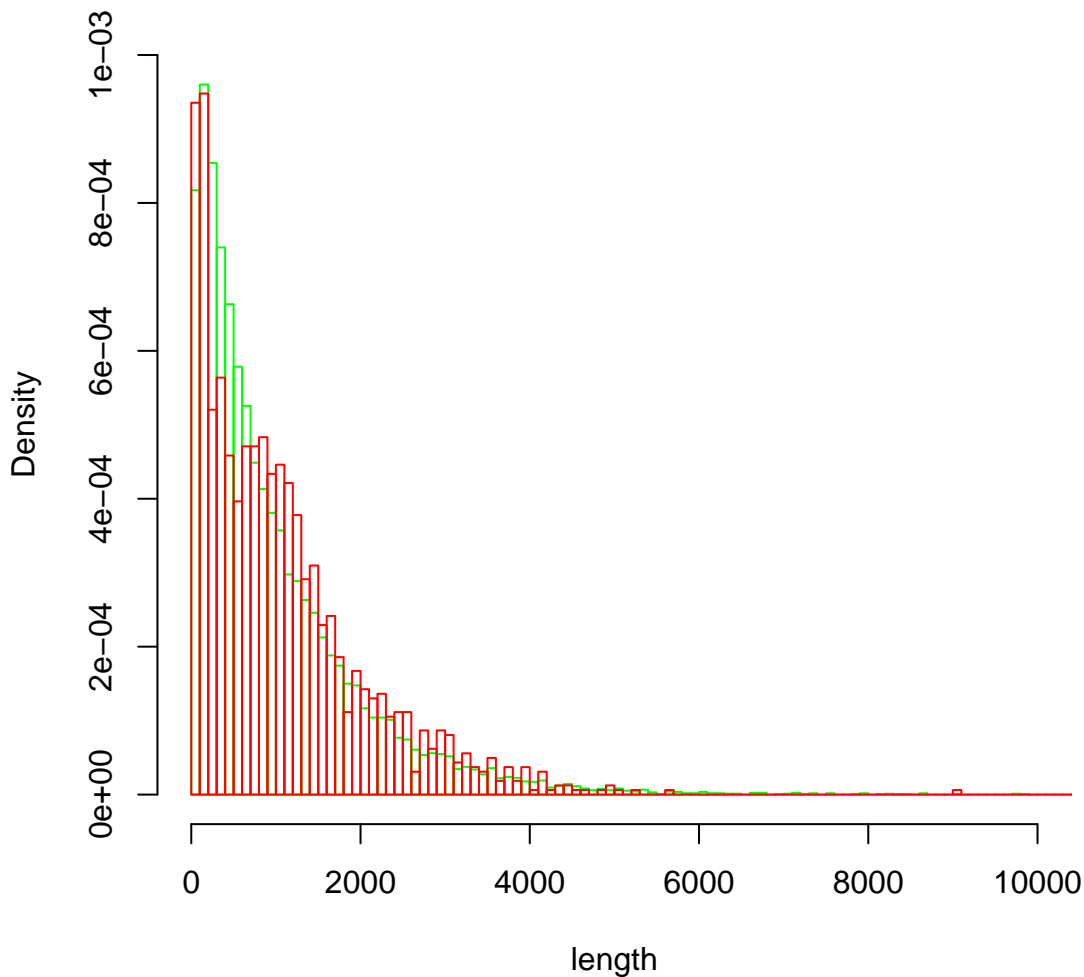

Supplement: Additional file 12 — Length distribution of 3' UTRs of the genes conserved overrepresented 7-mers. 3' UTR length of genes appearing in at least one of 465 sets selected by conserved overrepresentation (red) compared to all genes in our dataset (green). [file 1471-2105-8-174-S12.pdf]

## All genes vs conserved presence

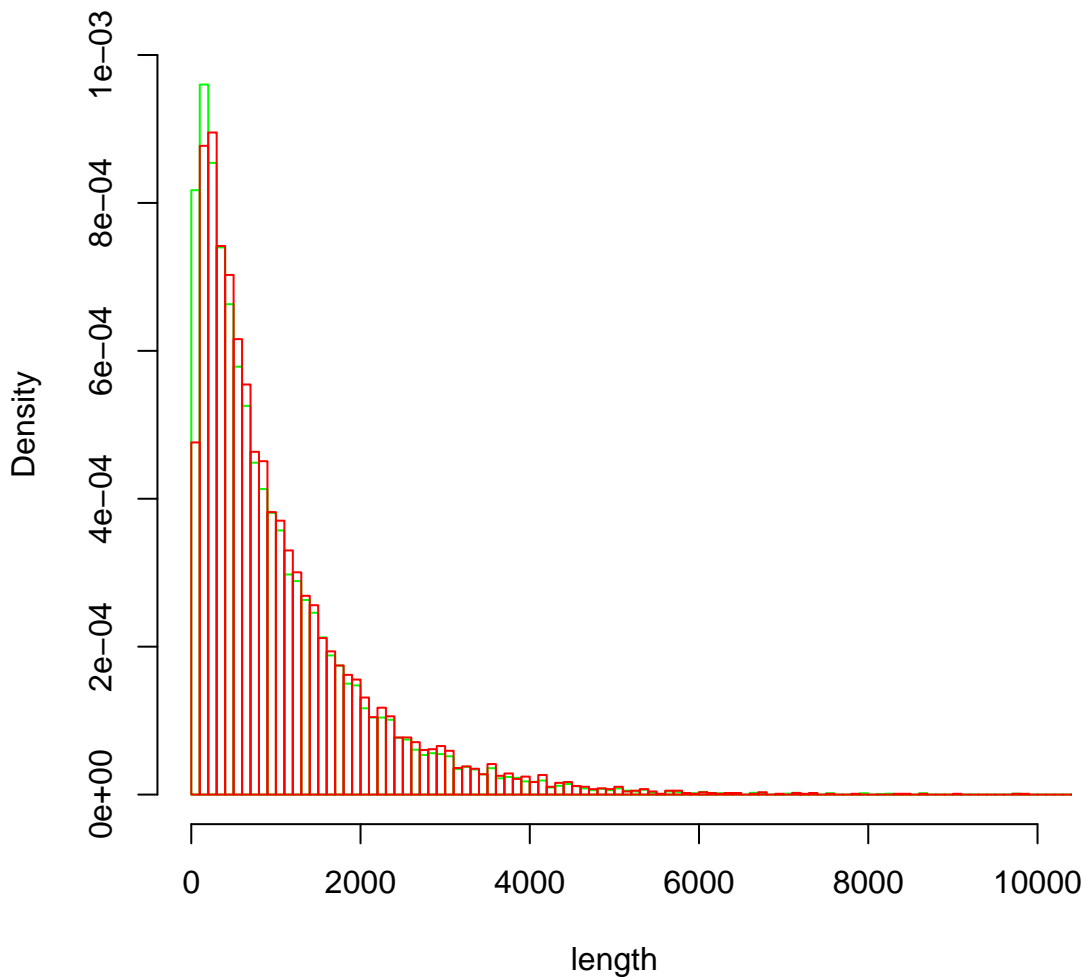

Supplement: Additional file 13 — Length distribution of 3' UTRs of the genes conserved instances of the 7-mers selected with the method of Ref [21]. The genes represented in red have at least one conserved instance of one of the 465 hightest-ranking 7-mers identified using the method introduced in [21]. The length distribution of their 3' UTRs is compared to the length distribution of all the genes in the dataset (green). [file 1471-2105-8-174-S13.pdf]
